# HybridGO-Loc: Mining Hybrid Features on Gene Ontology for Predicting Subcellular Localization of Multi-Location Proteins

**DOI:** 10.1371/journal.pone.0089545

**Published:** 2014-03-19

**Authors:** Shibiao Wan, Man-Wai Mak, Sun-Yuan Kung

**Affiliations:** 1 Department of Electronic and Information Engineering, The Hong Kong Polytechnic University, Hong Kong SAR, China; 2 Department of Electrical Engineering, Princeton University, Princeton, New Jersey, United States of America; Semmelweis University, Hungary

## Abstract

Protein subcellular localization prediction, as an essential step to elucidate the functions *in vivo* of proteins and identify drugs targets, has been extensively studied in previous decades. Instead of only determining subcellular localization of single-label proteins, recent studies have focused on predicting both single- and multi-location proteins. Computational methods based on Gene Ontology (GO) have been demonstrated to be superior to methods based on other features. However, existing GO-based methods focus on the occurrences of GO terms and disregard their relationships. This paper proposes a multi-label subcellular-localization predictor, namely HybridGO-Loc, that leverages not only the GO term occurrences but also the inter-term relationships. This is achieved by hybridizing the GO frequencies of occurrences and the semantic similarity between GO terms. Given a protein, a set of GO terms are retrieved by searching against the gene ontology database, using the accession numbers of homologous proteins obtained via BLAST search as the keys. The frequency of GO occurrences and semantic similarity (SS) between GO terms are used to formulate frequency vectors and semantic similarity vectors, respectively, which are subsequently hybridized to construct fusion vectors. An adaptive-decision based multi-label support vector machine (SVM) classifier is proposed to classify the fusion vectors. Experimental results based on recent benchmark datasets and a new dataset containing novel proteins show that the proposed hybrid-feature predictor significantly outperforms predictors based on individual GO features as well as other state-of-the-art predictors. For readers' convenience, the HybridGO-Loc server, which is for predicting virus or plant proteins, is available online at http://bioinfo.eie.polyu.edu.hk/HybridGoServer/.

## Introduction

Proteins located in appropriate physiological contexts within a cell are of paramount importance to exert their biological functions. Subcellular localization of proteins is essential to the functions of proteins and has been suggested as a means to maximize functional diversity and economize on protein design and synthesis [1]. Aberrant protein subcellular localization is closely correlated to a broad range of human diseases, such as Alzheimer's disease [2], kidney stone [3], primary human liver tumors [4], breast cancer [5], pre-eclampsia [6] and Bartter syndrome [7]. Knowing where a protein resides within a cell can give insights on drug targets identification and drug design [8], [9]. Wet-lab experiments such as fluorescent microscopy imaging, cell fractionation and electron microscopy are the gold standard for validating subcellular localization and are essential for the design of high quality localization databases such as The Human Protein Atlas (http://www.proteinatlas.org/). However, wet-lab experiments are time-consuming and laborious. With the avalanche of newly discovered protein sequences in the post-genomic era, computational methods are required to assist biologists to deal with large-scale proteomic data to determine the subcellular localization of proteins.

Conventionally, subcellular-localization predictors can be roughly divided into sequence-based and annotation-based. Sequence-based methods use (1) amino-acid compositions [10], [11], (2) sequence homology [12], [13], and (3) sorting signals [14], [15] as features. Annotation-based menthods use information beyond the protein sequences, such as Gene Ontology (GO) terms [16]–[21], Swiss-Prot keywords [22], and PubMed abstracts [23], [24]. A number of studies have demonstrated that methods based on GO information are superior to methods based on sequence-based features [25]–[28]. Note that the GO database contains not only experimental data but also predicted data (http://www.geneontology.org/GO.evidence.shtml), which may be determined by sequence-based methods. From this point of view, the GO-based prediction, which uses the GO annotation database to retrieve GO terms, is a filtering method for sequence-based predictions.

The GO comprises three orthogonal taxonomies whose terms describe the cellular components, biological processes, and molecular functions of gene products. The GO terms in each taxonomy are organized within a directed acyclic graph. These terms are placed within structural relationships, of which the most important being the ‘is-a’ relationship (*parent* and *child*) and the ‘part-of’ relationship (*part* and *whole*) [29], [30]. Recently, the GO consortium has been enriched with more structural relationships, such as ‘positively-regulates’, ‘negatively-regulates’ and ‘has-part’ [31], [32]. These relationships reflect that the GO hierarchical tree for each taxonomy contains redundant information, for which semantic similarity over GO terms can be found.

Instead of only determining subcellular localization of single-label proteins, recent studies have been focusing on predicting both single- and multi-location proteins. Since there exist multi-location proteins that can simultaneously reside at, or move between, two or more subcellular locations, it is important to include these proteins in the predictors. Actually, multi-location proteins play important roles in some metabolic processes that take place in more than one cellular compartment, e.g., fatty acid *β*-oxidation in the peroxisome and mitochondria, and antioxidant defense in the cytosol, mitochondria and peroxisome [33].

Recently, several multi-label predictors based on GO have been proposed, including Plant-mPLoc [34], Virus-mPLoc [35], iLoc-Plant [36], iLoc-Virus [37], KNN-SVM [38], mGOASVM [39] and others [40], [41]. These predictors have demonstrated superiority over sequence-based methods. These predictors use the occurrences of the GO terms but do not take the semantic relationships between GO terms into account.

Since the relationship between GO terms reflects the association between different gene products, protein sequences annotated with GO terms can be compared on the basis of semantic similarity measures. The semantic similarity over GO has been extensively studied and have been applied to many biological problems, including protein function prediction [42], [43], subnuclear localization prediction [44], protein-protein interaction inference [45]–[47] and microarray clustering [48]. The performance of these predictors depends on whether the similarity measure is relevant to the biological problems. Over the years, a number of semantic similarity measures have been proposed, some of which have been used in natural language processing.

Semantic similarity measures can be applied at the GO-term level or the gene-product level. At the GO-term level, methods are roughly categorized as node-based and edge-based. The node-based measures basically rely on the concept of information content of terms, which was proposed by Resnik [49] for natural language processing. Later, Lord et al. [50] applied this idea to measure the semantic similarity among GO terms. Lin et al. [51] proposed a method based on information theory and structural information. Subsequently, more node-based measures [52]–[54] were proposed. Edge-based measures are based on using the length or the depth of different paths between terms and/or their common ancestors [55]–[58]. At the gene-product level, two most common methods are pairwise approaches [59]–[63] and groupwise approaches [64]–[67]. Pairwise approaches measure similarity between two gene products by combining the semantic similarities between their terms. Groupwise approaches, on the other hand, directly group the GO terms of a gene product as a set, a graph or a vector, and then calculate the similarity by set similarity techniques, graph matching techniques or vector similarity techniques. More recently, Pesquita et al. [68] reviewed the semantic similarity measures applied to biomedical ontologies, and Guzzi et al. [69] provides a comprehensive review on the relationship between semantic similarity measures and biological features.

This paper proposes a multi-label predictor based on hybridizing frequency of occurrences of GO terms and semantic similarity between the terms for protein subcellular localization prediction. Compared to existing multi-label subcellular-localization predictors, our proposed predictor has the following advantages: (1) it formulates the feature vectors by hybridizing GO frequency of occurrences and GO semantic similarity features which contain richer information than only GO term frequencies; (2) it adopts a new strategy to incorporate richer and more useful homologous information from more distant homologs rather than using the top homologs only; (3) it adopts an adaptive decision strategy for multi-label SVM classifiers so that it can effectively deal with datasets containing both single-label and multi-label proteins. Results on two recent benchmark datasets and a new dataset containing novel proteins demonstrate that these three properties enable the proposed predictor to accurately predict multi-location proteins and outperform several state-of-the-art predictors.

## Methods

### Legitimacy of Using GO Information

Despite their good performance, GO-based methods have received some criticisms from the research community. The main argument of these criticisms is that the cellular component GO terms already have the cellular component categories, i.e., if the GO terms are known, the subcelluar locations will also be known. The prediction problem can therefore be easily solved by creating a lookup table using the cellular component GO terms as the keys and the cellular component categories as the hashed values. Such a naive solution, however, will lead to very poor prediction performance, as demonstrated and explained in our previous studies [28], [39]. A number of studies [70]–[72] by other groups also strongly support the legitimacy of using GO information for subcellular localization. For example, as suggested by [72], the good performance of GO-based methods is due to the high representation power of the GO space as compared to the Euclidean feature spaces used by the conventional sequence-based methods.

### Retrieval of GO Terms

The proposed predictor can use either the accession numbers (AC) or amino acid (AA) sequences of query proteins as input. Specifically, for proteins with known ACs, their respective GO terms are retrieved from the Gene Ontology annotation (GOA) database (http://www.ebi.ac.uk/GOA) using the ACs as the searching keys. For proteins without ACs, their AA sequences are presented to BLAST [73] to find their homologs, whose ACs are then used as keys to search against the GOA database.

**Figure 1:**
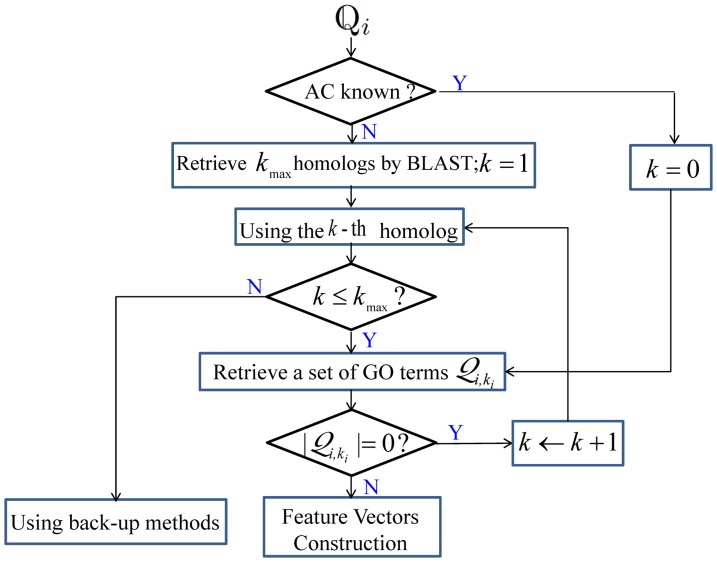
Procedures of retrieving GO terms. 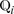
: the *i*-th query protein; 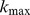: the maximum number of homologs retrieved by BLAST with the default parameter setting; 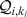: the set of GO terms retrieved by BLAST using the 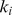-th homolog for the *i*-th query protein 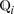; 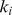: the 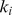-th homolog used to retrieve the GO terms.

While the GOA database allows us to associate the AC of a protein with a set of GO terms, for some novel proteins, neither their ACs nor the ACs of their top homologs have any entries in the GOA database; in other words, no GO terms can be retrieved by using their ACs or the ACs of their top homologs. In such case, the ACs of the homologous proteins, as returned from BLAST search, will be successively used to search against the GOA database until a match is found. With the rapid progress of the GOA database, it is reasonable to assume that the homologs of the query proteins have at least one GO term [17]. Thus, it is not necessary to use back-up methods to handle the situation where no GO terms can be found. The procedures are outlined in Fig 1.

### GO Frequency Features

Let 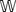 denote a set of distinct GO terms corresponding to a data set. 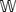 is constructed in two steps: (1) identifying all of the GO terms in the dataset and (2) removing the repetitive GO terms. Suppose 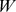 distinct GO terms are found, i.e., 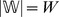; these GO terms form a GO Euclidean space with 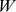 dimensions. For each sequence in the dataset, a GO vector is constructed by matching its GO terms against 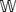, using the number of occurrences of individual GO terms in 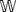 as the coordinates. Specifically, the GO vector 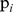 of the 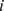-th protein 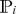 is defined as: 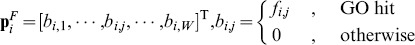
(1)where 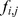 is the number of occurrences of the 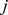-th GO term (term-frequency) in the 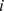-th protein sequence. The rationale is that the term-frequencies contain important information for classification. Note that 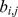
's are analogous to the term-frequencies commonly used in document retrieval.

Similarly, for the 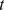-th query protein 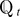, the GO frequency vector is defined as: 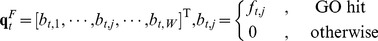
(2)


In the following sections, we use the superscript *F* to denote the GO frequency features in Eq. 2.

### Semantic-Similarity Features

Semantic similarity (SS) is a measure for quantifying the similarity between categorical data (e.g., words in documents), where the notion of similarity is based on the likeliness of meanings in the data. It is originally developed by Resnik [49] for natural language processing. The idea is to evaluate semantic similarity in an ‘is-a’ taxonomy using the shared information contents of categorical data. In the context of gene ontology, the semantic similarity between two GO terms is based on their most specific common ancestor in the GO hierarchy. The relationships between GO terms in the GO hierarchy, such as ‘is-a’ ancestor-child, or ‘part-of’ ancestor-child can be obtained from the SQL database through the link: http://archive.geneontology.org/latest-termdb/go_daily-termdb-tables.tar.gz. Note here only the ‘is-a’ relationship is considered for semantic similarity analysis [51]. Specifically, the semantic similarity between two GO terms *x* and *y* is defined as [49]: 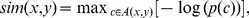
(3)where *A*(*x, y*) is the set of ancestor GO terms of both *x* and *y*, and *p*(*c*) is the probability of the number of gene products annotated to the GO term *c* divided by the total number of gene products annotated in the GO taxonomy.

While Resnik's measure is effective in quantifying the shared information between two GO terms, it ignores the distance between the terms and their common ancestors in the GO hierarchy. To further incorporate structural information from the GO hierarchy into the similarity measure, we have explored three extension of Resnik's measure, namely Lin's measure [51], Jiang's measure [74], and relevance similarity (RS) [52].

Given two GO terms *x* and *y*, the similarity by Lin's measure is: 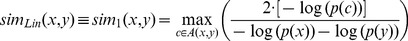
(4)


The similarity by Jiang's measure is: 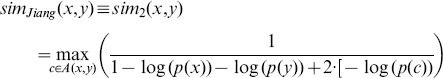
(5)


The similarity by RS is calculated as: 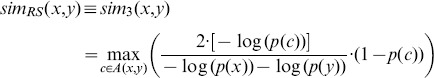
(6)


Among the three measures, 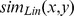 and 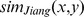 are relative measures that are proportional to the difference in information content between the terms and their common ancestors, which is independent of the absolute information content of the ancestors. On the other hand, 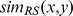 incorporates the probability of annotating the common ancestors as a weighing factor to Lin's measure. To simplify notations, we refer 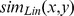, 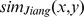 and 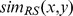 as 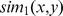, 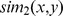 and 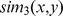, respectively.

Based on the semantic similarity between two GO terms, we adopted a continuous measure proposed in [48] to calculate the similarity between two proteins. Specifically, given two proteins 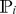 and 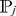, we retrieved their corresponding GO terms 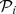 and 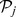 as described in the subsection “Retrieval of GO Terms”. (Note that strictly speaking, 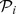 should be 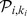, where 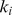 is the 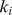-th homolog used to retrieve the GO terms for the 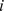-th protein. To simplify notations, we write it as 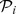.) Then, we computed the semantic similarity between two sets of GO terms 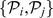 as follows: 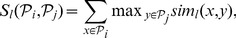
(7)where 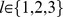, and 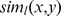 is defined in Eq. 4 to Eq. 6. 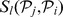 is computed in the same way by swapping 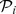 and 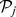. Finally, the overall similarity between the two proteins is given by: 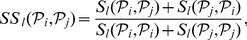
(8)where 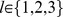. In the sequel, we refer the SS measures by Lin, Jiang and RS to as SS1, SS2 and SS3, respectively.

Thus, for a testing protein 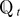 with GO term set 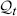, a GO semantic similarity (SS) vector 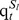 can be obtained by computing the semantic similarity between 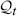 and each of the training protein 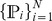, where 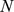 is the number of training proteins. Thus, 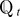 can be represented by an 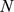-dimensional vector: 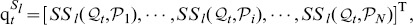
(9)where 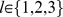. In other words, 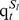 represents the SS vector by using the 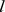-th SS measure.

### Hybridization of Two GO Features

As can be seen from the subsections “GO Frequency Features” and “Semantic-Similarity Features”, we know that the GO frequency features (Eq. 2) use the frequency of occurrences of GO terms, while GO SS features (Eq. 4 to Eq. 6) use the semantic similarity between GO terms. These two features are developed from two different perspectives. It is therefore reasonable to believe that these two kinds of information complement each other. Based on this assumption, we combine these two GO features and form a hybridized vector as: 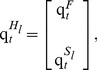
(10)where 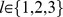. In other words, 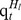 represents the hybridizing-feature vector by combining the GO frequency features and the SS features derived from the 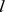-th SS measure. We refer them to as *Hybrid1*, *Hybrid2* and *Hybrid3*, respectively.

### Multi-label Multi-class SVM Classification

The hybridized-feature vectors obtained from the previous subsection are used for training multi-label one-vs-rest support vector machines (SVMs). Specifically, for an 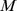-class problem (here 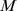 is the number of subcellular locations), 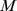 independent binary SVMs are trained, one for each class. Denote the hybrid GO vectors of the 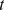-th query protein using the 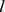-th SS measure as 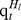. Given the 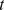-th query protein 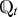, the score of the 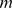-th SVM using the 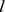-th SS measure is
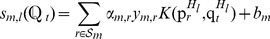
(11)where 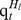 is the hybrid GO vector derived from 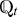 (See Eq. 10), 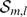 is the set of support vector indexes corresponding to the *m*-th SVM, 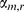 are the Lagrange multipliers, 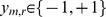 indicates whether the *r*-th training protein belongs to the *m*-th class or not, and 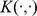 is a kernel function. Here, the linear kernel was used.

Unlike the single-label problem where each protein has one predicted label only, a multi-label protein could have more than one predicted labels. In this work, we compared two different decision schemes for this multi-label problem. In the first scheme, the predicted subcellular location(s) of the *i*-th query protein are given by
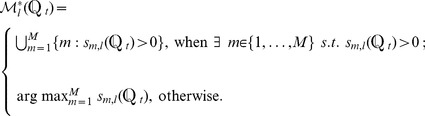
(12)


The second scheme is an improved version of the first one in that the decision threshold is dependent on the test protein. Specifically, the predicted subcellular location(s) of the *i*-th query protein are given by:

If 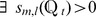,
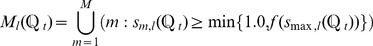
(13)otherwise, 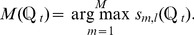
(14)


In Eq. 13, 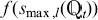 is a function of 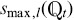, where 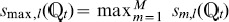. In this work, we used a linear function as follows: 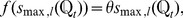
(15)where 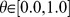 is a hyper-parameter that can be optimized through cross-validation.

In fact, besides SVMs, many other machine learning models, such as hidden Markov models (HMMs) and neural networks (NNs) [75], [76], have been used in protein subcellular-localization predictors. However, HMMs and NNs are not suitable for GO-based predictors because of the high dimensionality of GO vectors. The main reason is that under such condition, HMMs and NNs can be easily overtrained and thus lead to poor performance. On the other hand, linear SVMs can well handle high-dimensional data because even if the number of training samples is smaller than the feature dimension, linear SVMs are still able to find an optimal solution.

## Materials and Performance Metrics

### Datasets

In this paper, a virus dataset [35], [37] and a plant dataset [36] were used to evaluate the performance of the proposed predictor. The virus and the plant datasets were created from Swiss-Prot 57.9 and 55.3, respectively. The virus dataset contains 207 viral proteins distributed in 6 locations. Of the 207 viral proteins, 165 belong to one subcellular locations, 39 to two locations, 3 to three locations and none to four or more locations. This means that about 20% of the proteins in the dataset are located in more than one subcellular location. The plant dataset contains 978 plant proteins distributed in 12 locations. Of the 978 plant proteins, 904 belong to one subcellular locations, 71 to two locations, 3 to three locations and none to four or more locations. The sequence identity of both datasets was cut off at 25%.

**Figure 2:**
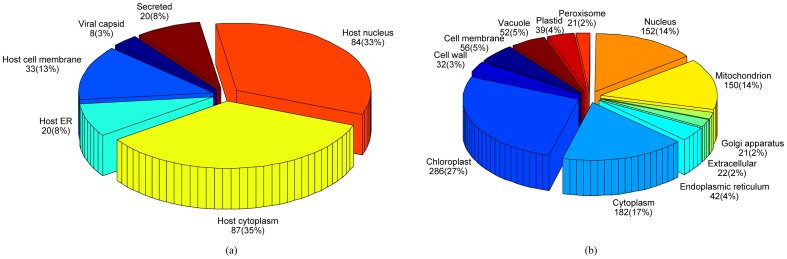
Breakdown of the (a) virus and (b) plant datasets. The number of proteins shown in each subcellular location represents the number of ‘locative proteins’ [37], [39]. For (a), there are 207 actual proteins and 252 locative proteins; For (b), there are 978 actual proteins and 1055 locative proteins.

The breakdown of these two datasets are listed in Figs. 2(a) and 2(b). Fig. 2(a) shows that the majority (68%) of viral proteins in the virus dataset are located in host cytoplasm and host nucleus while proteins located in the rest of the subcellular locations totally account only around one third. This means that this multi-label dataset is imbalanced across the six subcellular locations. Similar conclusions can be drawn from Fig. 2(b), where most of the plant proteins exist in chloroplast, cytoplasm, nucleus and mitochondrion while proteins in other 8 subcellular locations totally account for less than 30%. This imbalanced property makes the prediction of these two multi-label datasets difficult. These two benchmark datasets are downloadable from the hyperlinks in the HybridGO-Loc server.

### Performance Metrics

Compared to traditional single-label classification, multi-label classification requires more complicated performance metrics to better reflect the multi-label capabilities of classifiers. Conventional single-label measures need to be modified to adapt to multi-label classification. These measures include *Accuracy, Precision, Recall, F1-score (F1)* and *Hamming Loss (HL)* [77], [78]. Specifically, denote 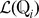 and 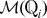 as the true label set and the predicted label set for the *i*-th protein 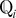 (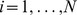), respectively. Here, 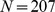 for the virus dataset and 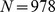 for the plant dataset. Then the five measurements are defined as follows:
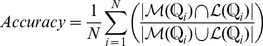
(16)

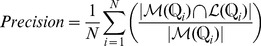
(17)

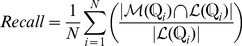
(18)

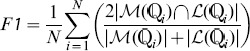
(19)

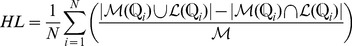
(20)where 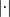 means counting the number of elements in the set therein and 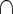 represents the intersection of sets.

*Accuracy, Precision, Recall* and *F1* indicate the classification performance. The higher the measures, the better the prediction performance. Among them, *Accuracy* is the most commonly used criteria. *F1-score* is the harmonic mean of *Precision* and *Recall*, which allows us to compare the performance of classification systems by taking the trade-off between *Precision* and *Recall* into account. The *Hamming Loss (HL)* [77], [78] is different from other metrics. As can be seen from Eq. 20, when all of the proteins are correctly predicted, i.e., 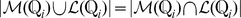 (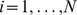), then 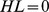; whereas, other metrics will be equal to 1. On the other hand, when the predictions of all proteins are completely wrong, i.e., 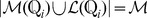 and 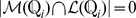, then 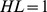; whereas, other metrics will be equal to 0. Therefore, the lower the *HL*, the better the prediction performance.

Two additional measurements [37], [39] are often used in multi-label subcellular localization prediction. They are overall locative accuracy (*OLA*) and overall actual accuracy (*OAA*). The former is given by: 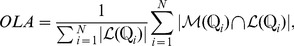
(21)and the overall actual accuracy (*OLA*) is: 
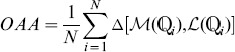
(22)where
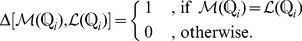
(23)


According to Eq. 21, a locative protein is considered to be correctly predicted if any of the predicted labels matches any labels in the true label set. On the other hand, Eq. 22 suggests that an actual protein is considered to be correctly predicted only if *all* of the predicted labels match those in the true label set exactly. For example, for a protein coexist in, say three subcellular locations, if only two of the three are correctly predicted, or the predicted result contains a location not belonging to the three, the prediction is considered to be incorrect. In other words, when and only when all of the subcellular locations of a query protein are exactly predicted without any overprediction or underprediction, can the prediction be considered as correct. Therefore, *OAA* is a more stringent measure as compared to *OLA*. *OAA* is also more objective than *OLA*. This is because locative accuracy is liable to give biased performance measures when the predictor tends to over-predict, i.e., giving large 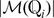 for many 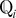. In the extreme case, if every protein is predicted to have all of the 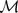 subcellular locations, according to Eq. 20, the *OLA* is 100%. But obviously, the predictions are wrong and meaningless. On the contrary, *OAA* is 0% in this extreme case, which definitely reflects the real performance.

Among all the metrics mentioned above, *OAA* is the most stringent and objective. This is because if only some (but not all) of the subcellular locations of a query protein are correctly predict, the numerators of the other 4 measures (Eqs. 16 to 21) are non-zero, whereas the numerator of *OAA* in Eq. 22 is 0 (thus contribute nothing to the frequency count).

In statistical prediction, there are three methods that are often used for testing the generalization capabilities of predictors: independent tests, sub-sampling tests (or 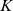-fold cross-validation) and leave-one-out cross validation (LOOCV). For independent tests, the selection of independent dataset often bears some sort of arbitrariness [79]; for the 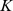-fold cross validation, different partitioning of a dataset will lead to different results, thus still being liable to statistical arbitrariness; for LOOCV, it will yield a unique outcome and is considered to be the most rigorous and bias-free method [80]. Hence, LOOCV was used to examine the performance of all predictors in this work. More detailed analysis of the statistical methods can be found in the supplementary materials. Note that the jackknife cross validation in iLoc-Plant and its variants is the same as LOOCV, as mentioned in [36], [79]. Because the term jackknife also refers to the methods that estimate the bias and variance of an estimator [81], to avoid confusion, we only use the term LOOCV in this paper.

## Results

### Comparing Different Features

**Figure 3:**
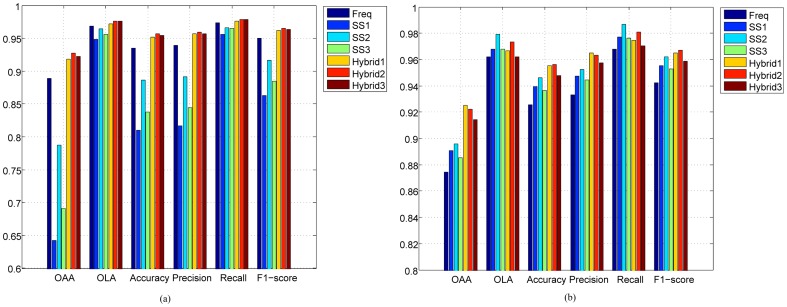
Performance of the hybrid features and individual features on the (a) virus and (b) plant datasets. *Freq*: GO frequency features; *SS1, SS2* and *SS3*: GO semantic similarity features by using Lin's measure [51], Jiang's measure [74] and RS measure [52], respectively; *Hybrid1, Hybrid2* and *Hybrid3*: GO hybrid features by combining GO frequency features with GO semantic similarity features based on *SS1, SS2* and *SS3*, respectively.

Fig. 3(a) shows the performance of individual and hybridized GO features on the virus dataset based on leave-one-out cross validation (LOOCV). In the figure, *SS1, SS2 and SS3* represent Lin's, Jiang's and RS similarity measures, respectively. *Hybrid1*, *Hybrid2* and *Hybrid3* represent the hybridized features obtained from these measures. As can be seen, in terms of all the six performance metrics, the performance of the hybrid features is remarkably better than the performance of individual features, regardless of which of the GO frequency features or the three GO SS features were used. Specifically, the *OAA*s (the most stringent and objective metric) of all of the three hybrid features are at least 3% (absolute) higher than that of the individual features, which suggests that hybridizing the two features can significantly boost the prediction performance. Moreover, among the hybridized features, the performance of *Hybrid2*, namely combining GO frequency features and GO SS features by Jiang's measure, outperforms *Hybrid1* and *Hybrid3*. Another interesting thing is that although all of the individual GO SS features perform much worse than the GO frequency features, the performance of the three hybridized features is still better that of any of the individual features. This suggests that the GO frequency features and SS features are complementary to each other.

Similar conclusions can be drawn from the plant dataset shown in Fig. 3(b). However, comparison between Fig. 3(a) and Fig. 3(b) reveals that for the plant dataset, the performance of hybridized features outperforms all of the individual features in terms of all metrics except *OLA* and *Recall*, while for the virus dataset, the former is superior to the latter in terms of all metrics. However, the losses in these two metrics do not outweigh the significant improvement on other metrics, especially on *OAA*, which has around 3% (absolute) improvement in terms of hybridized features as opposed to using individual features. Among the hybridizing features, *Hybrid2* also outperforms *Hybrid1* and *Hybrid3* in terms of *OLA, Accuracy, Recall* and *F1-score*, whereas *Hybrid1* performs better than others in terms of *OAA* and *Precision*. These results demonstrate that the GO SS features obtained by Lin's measure and Jiang's measure are better candidates than the RS measure for combining with the GO frequency features; however, there is no evidence suggesting which measure is better. It is also interesting to see that the performance of the three individual GO SS features is better than that of GO frequency features, in contrary to the results shown in Fig 3(a).

### Comparing with State-of-the-Art Predictors

Table 1 and Table 2 compare the performance of the proposed predictor against several state-of-the-art multi-label predictors on the virus and plant dataset based on leave-one-out cross validation. Note that we used the best performing hybridizing features with the adaptive decision strategy. Specifically, for both the virus and plant datasets, the best performance was achieved when *Hybrid2* and the adaptive decision strategy with 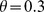 were used. 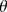 was determined by cross-validation as stated previously. Unless stated otherwise, we used *Hybrid2* to represent HybridGO-Loc in subsequent experiments. Our proposed predictor use the GO frequency features and GO semantic similarity features, whereas other predictors use only the GO frequency of occurrences as features. From the classification perspective, Virus-mPLoc [35] uses an ensemble OET-KNN (optimized evidence-theoretic K-nearest neighbors) classifier; iLoc-Virus [37] uses a multi-label KNN classifier; KNN-SVM [38] uses an ensemble of classifiers combining KNN and SVM; mGOASVM [39] uses a multi-label SVM classifier; and the proposed predictor use a multi-label SVM classifier incorporated with the adaptive decision scheme.

**Table 1:** Comparing the proposed predictor with state-of-the-art multi-label predictors based on leave-one-out cross validation (LOOCV) using the virus dataset.

Label	Subcellular Location	LOOCV Locative Accuracy (LA)				
		Virus-mPLoc [35]	KNN-SVM [38]	iLoc-Virus [37]	mGOASVM [39]	HybridGO-Loc
1	Viral capsid	8/8 = 1.000	8/8 = 1.000	8/8 = 1.000	8/8 = 1.000	8/8 = 1.000
2	Host cell membrane	19/33 = 0.576	27/33 = 0.818	25/33 = 0.758	32/33 = 0.970	32/33 = 0.970
3	Host ER	13/20 = 0.650	15/20 = 0.750	15/20 = 0.750	17/20 = 0.850	18/20 = 0.900
4	Host cytoplasm	52/87 = 0.598	86/87 = 0.988	64/87 = 0.736	85/87 = 0.977	85/87 = 0.966
5	Host nucleus	51/84 = 0.607	54/84 = 0.651	70/84 = 0.833	82/84 = 0.976	82/84 = 0.988
6	Secreted	9/20 = 0.450	13/20 = 0.650	15/20 = 0.750	20/20 = 1.000	20/20 = 1.000
Overall Locative Accuracy (*OLA*)		152/252 = 0.603	203/252 = 0.807	197/252 = 0.782	244/252 = 0.968	245/252 = **0.972**
Overall Actual Accuracy (*OAA*)		–	–	155/207 = 0.748	184/207 = 0.889	194/207 = **0.937**
*Accuracy*		–	–	–	0.935	**0.961**
*Precision*		–	–	–	0.939	**0.965**
*Recall*		–	–	–	0.973	**0.976**
*F1*		–	–	–	0.950	**0.968**
*HL*		–	–	–	0.026	**0.016**

“–” means the corresponding references do not provide the results on the respective metrics. *Host ER*: Host endoplasmic reticulum.

**Table 2:** Comparing the proposed predictor with state-of-the-art multi-label predictors based on leave-one-out cross validation (LOOCV) using the plant dataset.

Label	Subcellular Location	LOOCV Locative Accuracy (LA)			
		Plant-mPLoc [34]	iLoc-Plant [36]	mGOASVM [39]	HybridGO-Loc
1	Cell membrane	24/56 = 0.429	39/56 = 0.696	53/56 = 0.946	51/56 = 0.911
2	Cell wall	8/32 = 0.250	19/32 = 0.594	27/32 = 0.844	28/32 = 0.875
3	Chloroplast	248/286 = 0.867	252/286 = 0.881	272/286 = 0.951	278/286 = 0.972
4	Cytoplasm	72/182 = 0.396	114/182 = 0.626	174/182 = 0.956	168/182 = 0.923
5	Endoplasmic reticulum	17/42 = 0.405	21/42 = 0.500	38/42 = 0.905	38/42 = 0.905
6	Extracellular	3/22 = 0.136	2/22 = 0.091	22/22 = 1.000	21/22 = 0.955
7	Golgi apparatus	6/21 = 0.286	16/21 = 0.762	19/21 = 0.905	19/21 = 0.905
8	Mitochondrion	114/150 = 0.760	112/150 = 0.747	150/150 = 1.000	149/150 = 0.993
9	Nucleus	136/152 = 0.895	140/152 = 0.921	151/152 = 0.993	150/152 = 0.987
10	Peroxisome	14/21 = 0.667	6/21 = 0.286	21/21 = 1.000	21/21 = 1.000
11	Plastid	4/39 = 0.103	7/39 = 0.179	39/39 = 1.000	38/39 = 0.974
12	Vacuole	26/52 = 0.500	28/52 = 0.538	49/52 = 0.942	48/52 = 0.923
Overall Locative Accuracy (*OLA*)		672/1055 = 0.637	756/1055 = 0.717	1015/1055 = **0.962**	1009/1055 = 0.956
Overall Actual Accuracy (*OAA*)		–	666/978 = 0.681	855/978 = 0.874	915/978 = **0.936**
*Accuracy*		–	–	0.926	**0.959**
*Precision*		–	–	0.933	**0.972**
*Recall*		–	–	**0.968**	**0.968**
*F1*		–	–	0.942	**0.966**
*HL*		–	–	0.013	**0.007**

“–” means the corresponding references do not provide the results on the respective metrics.

As shown in Table 1, the proposed predictor perform significantly better than the other predictors. The *OAA* and *OLA* of the proposed predictor are more than 15% (absolute) higher than that of iLoc-Virus and Virus-mPLoc. It also performs significantly better than KNN-SVM in terms of *OLA*. When comparing with mGOASVM, the proposed predictor performs remarkably better in of all of the performance metrics, especially for the *OAA* (0.937 vs 0.889). These results demonstrate that hybridizing the GO frequency features and GO SS features can significantly boost prediction performance, which also suggests that these two kinds of information are proved to be complementary to each other in terms of predicting subcellular localization. Similar conclusions can be drawn for the plant dataset from Table 2 except that the *OLA* of the proposed predictor is slightly worse than that of mGOASVM, and the *Recall* is equivalent to that of mGOASVM. Nevertheless, the small losses do not outweigh the impressive improvement in the other metrics, especially in the *OAA* (0.936 vs 0.874).

### Prediction of Novel Proteins

To further demonstrate the effectiveness of HybridGO-Loc, a newer plant dataset constructed for mGOASVM [39] was used to compare with state-of-the-art multi-label predictors using independent tests. Specifically, this new plant dataset contains 175 plant proteins, of which 147 belong to one subcellular location, 27 belong to two locations, 1 belong to three locations and none to four or more locations. These plant proteins were added to Swiss-Prot between 08-Mar-2011 and 18-Apr-2012. Because the plant dataset used for training the predictors was created on 29-Apr-2008, there is an almost 3-year time gap between the training data and test data in our experiments.

Table 3 compare the performance of HybridGO-Loc against several state-of-the-art multi-label plant predictors on the new plant dataset. All the predictors use the 978 proteins of the plant dataset (See Fig. 2(b)) for training the classifier and make independent test on the new 175 proteins. As can be seen, HybridGO-Loc performs significantly better than all the other predictors in terms of all of the performance metrics. Similar conclusions can also be drawn from the performance in individual subcellular locations.

**Table 3:** Comparing HybridGO-Loc with state-of-the-art multi-label plant predictors based on independent tests using the new plant dataset.

Label	Subcellular Location	Independent Test Locative Accuracy			
		Plant-mPLoc [34]	iLoc-Plant [36]	mGOASVM [39]	HybridGO-Loc
1	Cell membrane	8/16 = 0.500	1/16 = 0.063	7/16 = 0.438	16/16 = 1.000
2	Cell wall	0/1 = 0	0/1 = 0	0/1 = 0%	1/1 = 1.000
3	Chloroplast	27/54 = 0.500	45/54 = 0.833	39/54 = 0.722	30/54 = 0.556
4	Cytoplasm	5/38 = 0.132	15/38 = 0.395	19/38 = 0.500	31/38 = 0.816
5	Endoplasmic reticulum	1/9 = 0.111	1/9 = 0.111	3/9 = 0.333	4/9 = 0.444
6	Extracellular	0/3 = 0	0/3 = 0	1/3 = 0.333	0/3 = 0
7	Golgi apparatus	3/7 = 0.429	1/7 = 0.143	3/7 = 0.429	7/7 = 1.000
8	Mitochondrion	6/16 = 0.375	3/16 = 0.188	11/16 = 0.688	16/16 = 1.000
9	Nucleus	31/46 = 0.674	43/46 = 0.935	33/46 = 0.717	44/46 = 0.957
10	Peroxisome	4/6 = 0.667	0/6 = 0	3/6 = 0.500	4/6 = 0.667
11	Plastid	0/1 = 0	0/1 = 0	0/1 = 0	0/1 = 0
12	Vacuole	2/7 = 0.286	4/7 = 0.571	4/7 = 0.571	7/7 = 1.000
Overall Locative Accuracy (*OLA*)		87/204 = 0.427	113/204 = 0.554	123/204 = 0.603	160/204 = **0.784**
Overall Actual Accuracy (*OAA*)		60/175 = 0.343	91/175 = 0.520	97/175 = 0.554	127/175 = **0.726**
*Accuracy*		0.417	0.574	0.594	**0.784**
*Precision*		0.444	0.626	0.630	**0.826**
*Recall*		0.474	0.577	0.609	**0.798**
*F1*		0.444	0.592	0.611	**0.803**
*HL*		0.116	0.076	0.075	**0.037**

**Figure 4:**
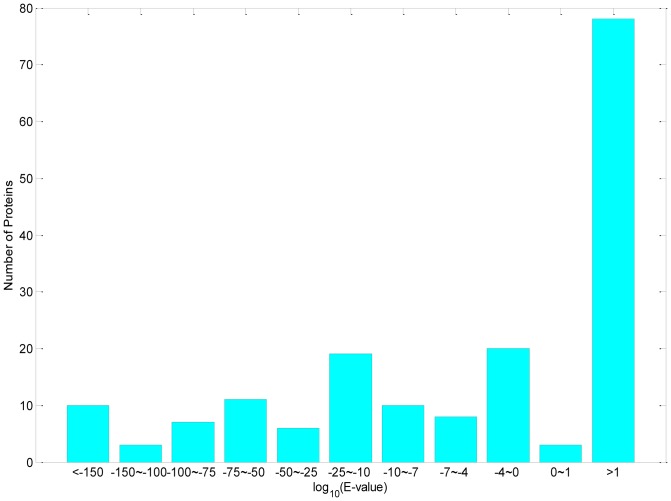
Distribution of the closeness between the new testing proteins and the training proteins. The *closeness* is defined as the BLAST E-values of the training proteins using the test proteins as the query proteins in the BLAST searches. *Number of Proteins*: The number of testing proteins whose E-values fall into the interval specified under the bar. Small E-values suggest that the corresponding new proteins are close homologs of the training proteins.

Fig. 4 shows the distribution of the E-values of the test proteins, which were obtained by using the training proteins as the repository and the test proteins as the query proteins in the BLAST search. If we use a common criteria that homologous proteins should have E-value less than 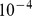, then 74 out of 175 test proteins are homologs of the training proteins, which account for 42% of the test set. Note that this homologous relationship does not mean that using BLAST's homology transfers can predict all of the 74 test proteins correctly. In fact, BLAST's homology transfers (based on the CC field of the homologous proteins) can only achieve a prediction accuracy of 26.9% (47/175). As the prediction accuracy of HybridGO-Loc on this test set (see Table 3) is significantly higher than this percentage, the extra information available from the GOA database plays a very important role in the prediction.

## Discussion

### Semantic Similarity Measures

In this paper, we have compared three of the most common semantic similarity measures for subcellular localization, including Lin's measure [51], Jiang's measure [74], and relevance similarity measure [52]. We excluded Resnik's measure because it ignores the distance between the terms and their common ancestors in the GO hierarchy. In addition to these measures, many online tools are also available for computing the semantic similarity at the GO-term level and gene-product level [44], [82]–[84]. However, these measures are discrete measures whereas the measures that we used are continuous. Research has shown that continuous measures are better than discrete measures in many applications [48].

### GO-Frequency Features versus SS Features

Note that we do not replace the GO frequency vectors. Instead, we augment the GO frequency feature with a more sophisticated feature, i.e. the GO SS vectors, which are to be combined with the GO frequency vectors. A GO frequency vector is found by counting the number of occurrences of every GO term in a set of distinct GO terms obtained from the training dataset, whereas an SS vector is constructed by computing the semantic similarity between a test protein with each of the training proteins at the gene-product level. That is, each element in an SS vector represents the semantic similarity of two GO-term groups. This can be easily seen from their definitions in Eq. 2 and Eq. 4–9, respectively.

The GO frequency vectors and the GO SS vectors are different in two fundamental ways.

- A). GO frequency vectors are more *primitive* in the sense that their elements are based on individual GO terms without considering the inter-term relationship, i.e., the elements in a GO frequency vectors are independent of each other.
- B). GO SS vectors are more *sophisticated* in the following two senses:
  - B1) *Inter-term relationship*. SS vectors are based on inter-term relationships. They are defined on a space in which each basis corresponds to one training protein and the coordinate along that basis is defined by the semantic similarity between a testing protein and the corresponding training protein.
  - B2) *Inter-group relationship*. The pairwise relationships between a test protein and the training proteins are hierarchically structured. This is because each basis of the SS space depends on a group of GO terms of the corresponding training protein, and the terms are arranged in a hierarchical structure (parent-child relationship). Because the GO terms in different groups are not mutually exclusive, the bases in the SS space are not independent of each other.

### Bias Analysis

Except for the new plant dataset, we adopted LOOCV to examine the performance of all predictors in this work, which is considered to be the most rigorous and bias-free [80]. Nevertheless, determining the set of distinct GO terms 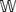 from a dataset is by no means without bias, which may favor the LOOCV performance. This is because the set of distinct GO terms 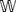 derived from a given dataset may not be representative for other datasets; in other words, the generalization capabilities of the predictors may be weakened when new GO terms outside 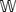 are found in the test proteins.

However, we have the following strategies to minimize the bias. First, the two benchmark datasets used in this paper were constructed based on the whole Swiss-Prot database (although in different years), which, to some extent, incorporated all the possible information of plant proteins or virus proteins in the database. In other words, 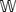 was constructed based on all of the GO terms corresponding to the whole Swiss-Prot database, which enables 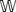 to be representative for all of the distinct GO terms. Second, these two benchmark datasets were collected according to strict criteria. Details of the procedures can be found in the supplementary materials. and the sequence similarity of both datasets was cut off at 25%, which enables us to use a small set of representative proteins to represent all of the proteins of the corresponding species (i.e., virus or plant) in the whole database. In other words, 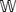 will vary from species to species, yet still be statistically representative for all of the useful GO terms for the corresponding species. Third, using 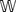 for statistical performance evaluation is equivalent or at least approximate to using all of the distinct GO terms in the GOA database. This is because other GO terms that do not correspond to the training proteins will not participate in training the linear SVMs, nor will they play essential roles in contributing to the final predictions. In other words, the generalization capabilities of HybridGO-Loc will not be weakened even if some new GO terms are found in the test proteins. A mathematical proof of this statement can be found in the supplementary materials available in the HybridGO-Loc server.

One may argue that the performance bias might arise when the whole 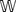 was used to construct the hybrid GO vectors for both training and testing during cross validation. This is because, in each fold of the LOOCV, the training proteins and the singled-out test protein will use the same 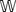 to construct the GO vectors, meaning that the SVM training algorithm can *see* some information of the test protein indirectly through the GO vector space defined by 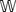. It is possible that for a particular fold of LOOCV, the GO terms of a test protein do not exist in any of the training proteins. However, we have mathematically proved that this bias will not exist during LOOCV (see the accompanying supplementary materials for the proof). Furthermore, the results of the independent tests (See Table 3) for which no such bias occurs also strongly suggest that HybridGO-Loc outperforms other predictors by a large margin.

## Conclusions

This paper proposes a new multi-label predictor by hybridizing GO frequency features and semantic similarity features to predict the subcellular locations of multi-label proteins. Three different semantic similarity measures have been investigated to be combined with GO frequency features to formulate GO hybrid feature vectors. The feature vectors are subsequently recognized by multi-label multi-class support vectors machine (SVM) classifiers equipped with an adaptive decision strategy that can produce multiple class labels for a query protein. Compared to existing multi-label subcellular-localization predictors, our proposed predictor has the following advantages: (1) it formulates the feature vectors by hybridizing GO frequency of occurrences and GO semantic similarity features which contains richer information than only GO term frequencies; (2) it adopts a new strategy to incorporate richer and more useful homologous information from more distant homologs rather than using the top homologs only; (3) it adopts an adaptive decision strategy for multi-label SVM classifiers so that it can effectively deal with datasets containing both single-label and multi-label proteins. Experimental results demonstrate the superiority of the proposed hybrid features over each individual features. It was also found that the proposed predictor performs remarkably better than existing state-of-the-art predictors. For readers' convenience, HybridGO-Loc is available online at http://bioinfo.eie.polyu.edu.hk/HybridGoServer/.
